# The Impact of Coronavirus Disease 2019 on Sexual Health

**DOI:** 10.31661/gmj.v9i0.1928

**Published:** 2020-12-26

**Authors:** Mojdeh Banaei, Vida Ghasemi, Mohammad Dordeh, Nasibeh Roozbeh

**Affiliations:** ^1^Mother and Child Welfare Research Center, Hormozgan University of Medical Sciences, Bandar Abbas, Iran; ^2^Student Research Committee, Department of Midwifery and Reproductive Health, School of Nursing and Midwifery, Shahid Beheshti University of Medical Sciences, Tehran, Iran; ^3^Asadabad Faculty of Medical Sciences, Asadabad, Iran; ^4^Department of Psychology, Bandar Abbas Branch, Islamic Azad University, Bandar Abbas, Iran

**Keywords:** COVID-19, SARS-CoV-2, Sexual Behavior, Sexual Health

## Dear Editor,

During marital life, numerous variables affect how couples communicate with each other, and these variables can lead to the satisfaction or dissatisfaction of the couple with the marital relationship [1]. Some of these variables include income, employment status, number of children, sexual satisfaction, and some diseases [2]. Since late December 2019, a new coronavirus disease called COVID-19 has been reported in Wuhan, China, which has subsequently affected several countries around the world [3]. Increased prevalence of COVID-19 increased not only public health concerns but also caused psychological distress in individuals [4]. Studies in China have reported that both the medical staff and members of the community experienced psychological problems, including anxiety, depression, and stress, following an increase in the number of confirmed cases and deaths from COVID-19 [5,6]. In the severe acute respiratory syndrome (SARS) epidemic (2003) in Hong Kong, Lau *et al*. showed that financial stress was associated with worsening sexual function and mental health, and 6.1% of the participants reported poor sexual function [7]. However, the evidence suggests that the novel coronavirus epidemics are also associated with economic anxiety [8]. On the other hand, the pandemic of infection and the implementation of home isolation programs in some countries can cause psychological symptoms such as post-traumatic stress, economic damage, fear and anxiety, and anger in individuals [9]. All of these can affect people’s sexual health, and these problems may continue for a long time after the eradication of infection. The results of a study in the SARS epidemic showed that about a year after the epidemic, people experienced high levels of stress and had symptoms of anxiety, depression, and post-traumatic stress [10]. Reducing a person’s mental health by reducing interpersonal communication in various aspects of life can have a negative impact on the quality of married life, which is the foundation of family health [11]. Iran, like other countries, has been severely affected by the COVID-19 pandemic, and this pandemic can affect different domains of couples′s sexual function. Therefore, we reported the results of the pilot study that performed to survey the sexual health of women during the epidemic phases of COVID-19. In a cross-sectional survey, about 130 married women of childbearing age were evaluated. The mean duration of marriage in 12% of cases was less than one year; in 47% of them it was 1-5 years, and in 30% of them it was 10-16 years. Also, 12% of women are afraid of having sexual intercourse and 13% are afraid to have intimate interactions with their husbands; 22% of women reported that their sexual intercourse decreased, 67% have no change, and 11% increased, 34% and 31% of women reported that their sexual relationship was 1-4 and 5-8 times in a month, respectively. About 74% of women reported that their intimate relationships with their husbands did not change during the COVID-19 pandemic. About 77% of women reported that their sexual desires have no changes during this period, while about 17% of them reported that sexual desire of both themselves and their spouse were reduced. In the present study, in some participants in the COVID-19 pandemic, the sexual desire and the frequency of sexual intercourse was reduced, which could indicate the negative impact of this epidemic infection on the couple’s sexual health. Therefore, it is recommended that the sexologist and reproductive health professionals provide comprehensive sexual health care guidelines for pre-, during-, and post-crisis stages. Also, since psychological problems and, consequently, problems related to marital and sexual relationships during the COVID-19 epidemic crisis may persist for a long time after the infection is eradicated, it is recommended that sexual and reproductive health professionals take steps to improve Sexual health of couples.

## Acknowledgment

 This research was approved by the Ethics Committee of Hormozgan University of Medical Sciences (IR.HUMS.REC.1399.030). Hereby, we highly appreciate the cooperation of the personnel of Mother and Child Welfare Research Center and Hormozgan University of Medical Sciences

## Conflict of Interest

 The authors declare that they have no conflict of interest.
